# Identifying the Occurrence Time of the Deadly Mexico M8.2 Earthquake on 7 September 2017

**DOI:** 10.3390/e21030301

**Published:** 2019-03-20

**Authors:** Nicholas V. Sarlis, Efthimios S. Skordas, Panayiotis A. Varotsos, Alejandro Ramírez-Rojas, Elsa Leticia Flores-Márquez

**Affiliations:** 1 Section of Solid State Physics, Department of Physics, National and Kapodistrian University of Athens, Panepistimiopolis, 157 84 Zografos, Athens, Greece; 2 Solid Earth Physics Institute, Department of Physics, National and Kapodistrian University of Athens, Panepistimiopolis, 157 84 Zografos, Athens, Greece; 3 Departamento de Ciencias Básicas, Universidad Autónoma Metropolitana Azcapotzalco, Av. San Pablo 180, Mexico City 02200, Mexico; 4 Instituto de Geofísica, Universidad Nacional Autónoma de México, Mexico City 04510, Mexico

**Keywords:** natural time analysis, order parameter of seismicity, entropy change under time reversal, entropy, critical point

## Abstract

It has been shown that some dynamic features hidden in the time series of complex systems can be unveiled if we analyze them in a time domain termed natural time. In this analysis, we can identify when a system approaches a critical point (dynamic phase transition). Here, based on natural time analysis, which enables the introduction of an order parameter for seismicity, we discuss a procedure through which we could achieve the identification of the occurrence time of the M8.2 earthquake that occurred on 7 September 2017 in Mexico in Chiapas region, which is the largest magnitude event recorded in Mexico in more than a century. In particular, we first investigated the order parameter fluctuations of seismicity in the entire Mexico and found that, during an almost 30-year period, i.e., from 1 January 1988 until the M8.2 earthquake occurrence, they were minimized around 27 July 2017. From this date, we started computing the variance of seismicity in Chiapas region and found that it approached the critical value 0.070 on 6 September 2017, almost one day before this M8.2 earthquake occurrence.

## 1. Introduction

In the beginning of the 2000s, a new procedure for the study of complex time series, termed natural time analysis, was proposed [1,2,3,4]. This unveils unique dynamic features hidden behind the time series of complex systems and has found applications in diverse fields with encouraging results, which have been compiled in a monograph [5]. Natural time was considered by Turcotte and coworkers [6,7,8,9,10] as the basis for a new methodology (“nowcasting”) to estimate the seismic risk.

Seismicity is a typical example of complex time series and earthquakes exhibit complex correlations in time, space and magnitude (*M*) which have been the object of several studies (e.g., [11,12,13,14,15,16]). It is nowadays widely accepted that the observed earthquake scaling laws (e.g., [17]) indicate the existence of phenomena closely associated with the proximity of the system to a critical point [1,5,18,19]. Following this view that earthquakes are critical phenomena, we face the difficulty to identify an order parameter, which is a parameter by means of which we can determine when the system approaches the critical point. In general, the order parameter of a system in the critical state is expected to undergo non-Gaussian fluctuations, but almost nothing is known [20] about the mathematical form of the possible probability distributions of the order parameter except of a few cases [20,21]. Such an attempt to introduce an order parameter for seismicity has been made by means of natural time analysis.

In a time series comprising *N* earthquakes, the natural time $\chi_{k}=k/N$ serves as an index for the occurrence of the *k*th earthquake. This index together with the energy $Q_{k}$ released during the *k*th earthquake of magnitude $M_{k}$, i.e., the pair $\left(\chi_{k},Q_{k}\right)$, is studied in natural time analysis. One computes alternatively the pair $\left(\chi_{k},p_{k}\right)$, where

(1) $$p_{k}=\frac{Q_{k}}{\sum_{n=1}^{N}Q_{n}}$$

denotes the normalized energy released during the *k*th earthquake. The variance of χ weighted for $p_{k}$, labeled $\kappa_{1}$, is given by [1,3,4,5,22,23]

(2) $$\kappa_{1}=\sum_{k=1}^{N}p_{k}{\left(\chi_{k}\right)}^{2}-{\left(\sum_{k=1}^{N}p_{k}\chi_{k}\right)}^{2},$$

where $Q_{k}$, and hence $p_{k}$, for earthquakes is estimated through the relation [24]

(3) $$Q_{k}\propto 10^{1.5M_{k}}.$$

The quantity $\kappa_{1}$, the variance of natural time $\chi_{k}$, plays an important role in recognizing when the evolving complex dynamic system under study enters the critical stage [1,2,5]. This occurs when the variance $\kappa_{1}$ converges to 0.070, as discussed in detail in the Appendix of Reference [25]. In particular, in References [5,26], it is shown that $\kappa_{1}$ becomes equal to 0.070 at the critical state for a variety of dynamical models including the 2D Ising model and the self organized criticality (SOC) Bak–Tang–Wiesenfeld sandpile model. Further, in Table 8.1 of Reference [5], one can find a compilation of 14 cases in which the condition $\kappa_{1}$ = 0.070 has been ascertained. Specifically, in Reference [22], it is explained that the quantity $\kappa_{1}$ given by Equation (2) can be considered as an order parameter for seismicity since its value changes abruptly when a mainshock (the new phase) occurs, and in addition the statistical properties of its fluctuations resemble those in other non-equilibrium and equilibrium critical systems. Note that at least six earthquakes are needed for obtaining reliable $\kappa_{1}$ [22].

Upon considering a moving natural time window comprising *i* consecutive events sliding, event by event, through the earthquake catalog, the computed $\kappa_{1}$ values enable the calculation of their average value $\mu (\kappa_{1})$ and their standard deviation $\sigma (\kappa_{1})$. We then determine the variability β of $\kappa_{1}$, i.e., the quantity $\beta_{i}$ [27]

(4) $$\beta_{i}=\frac{\sigma (\kappa_{1})}{\mu (\kappa_{1})}.$$

that corresponds to this natural time window of length *i*. The time evolution of $\beta_{i}$ is followed by applying the procedure explained in References [5,23] as well as in References [28,29].

Recent investigations on the fluctuations of the order parameter of seismicity, based on natural time analysis, shows [30] that they exhibit a minimum $\beta_{min}$ upon the initiation of a series of precursory low frequency (≤0.1 Hz) electric signals, termed Seismic Electric Signals (SES) activity [31,32], as for example the one recorded by Uyeda et al. in 2000 in Japan [33,34]. Once a SES activity has been recorded, a few weeks to $5\frac{1}{2}$ months before a major EQ [5], the candidate epicentral area can be determined on the basis of the so-called selectivity map of the station at which the SES was recorded as well as by taking into account the ratio of the two SES components (e.g., [31,32]). The minimum $\beta_{min}$ of the fluctuations of the order parameter of seismicity before the $M_{W}$9 Tohoku earthquake that occurred on 11 March 2011 was observed [28] on 5 January 2011 and remarkably is the deepest minimum ever observed during the period 1984–2011 investigated. Interestingly, this date is almost simultaneous with the initiation of an SES activity—being in accordance with the findings in References [30]—since anomalous magnetic field variations appeared during the period 4–14 January 2011 on the z component at two measuring sites (Esashi and Mizusawa) lying at epicentral distances of around 130 km [35,36,37]. Subsequently, in Reference [29], it is shown that a spatiotemporal study of the minimum $\beta_{min}$ enables an estimate of the epicentral area of the impending major earthquake (without making use of SES data) by means of the following procedure [29]: By dividing the entire Japanese region N${}_{25}^{46}$E${}_{125}^{148}$ (large area) into small areas, a calculation of the fluctuations of $\kappa_{1}$ of seismicity is carried out on them. Some small areas show a minimum of the fluctuations almost simultaneously with the minimum $\beta_{min}$ in the entire Japanese region and such small areas cluster within a few hundred km from the actual epicenter. Such a calculation for the aforementioned $M_{W}$9 Tohoku earthquake led to an estimate of the candidate epicentral area. Following the same procedure, the candidate epicentral areas for all shallow mainshocks of magnitude 7.6 or larger in Japan during 1984–2011 have been estimated [29].

Very recently [25] the procedure through which the occurrence time of an impending major earthquake can be determined has been reviewed. Such a determination can be achieved in principle by analyzing in natural time the seismicity in the candidate area (e.g., see References [1,26]). To apply this general procedure, there exist however two important prerequisites: First, it is necessary to know when to start the analysis, i.e., set the natural time for seismicity equal to zero. This is the time at which the system enters the critical stage. Second, a reliable estimation of the candidate epicentral area is needed. If geoelectrical measurements are carried out, both these prerequisites are met upon the recording of an SES activity, because its initiation marks the time when the system enters the critical stage [23] (see also Section 3) and the SES data enable the determination of the epicentral area of the impending mainshock [31,32], as already mentioned. On the other hand, if geoelectrical data are lacking, we take advantage of the following two findings resulting from natural time analysis of seismicity mentioned above: First, the fluctuations of the order parameter of seismicity in a large area exhibit a minimum $\beta_{min}$ a few months before a major earthquake almost simultaneously with the initiation of an SES activity [30]. Second, a spatiotemporal study of the minimum $\beta_{min}$ unveils an estimate of the epicentral area of the impending major earthquake [29]. These two findings are used, for example, in Reference [25] for the determination of the occurrence time of the $M_{W}$9 Tohoku earthquake (EQ) in Japan on 11 March 2011 as follows: By starting from 5 January 2011 (recall that this is the date of $\beta_{min}$ mentioned above), we computed the $\kappa_{1}$ values in the candidate epicentral area determined in Reference [29]. We then found that the condition $\kappa_{1}=0.070$ [26] (which signals that the mainshock is going to occur within the next few days or so) was fulfilled for $M_{thres}$= 4.2–5.0 in the morning of 10 March 2011 upon the occurrence of the earthquakes from 08:36 to 13:14 LT, i.e., almost one day before the Tohoku EQ (see the gray shaded area in Figure 6b of Reference [25]). This result showing that the critical point has been approached almost one day before the Tohoku EQ occurrence, i.e., in the morning of 10 March 2011, is very important since it revealed that natural time analysis enabled the recognition that the approach to the critical point (mainshock) happened almost a day after the occurrence of the *M*7.3 earthquake of 9 March 2011, thus recognizing that this *M*7.3 earthquake was a foreshock.

**Figure 1:**
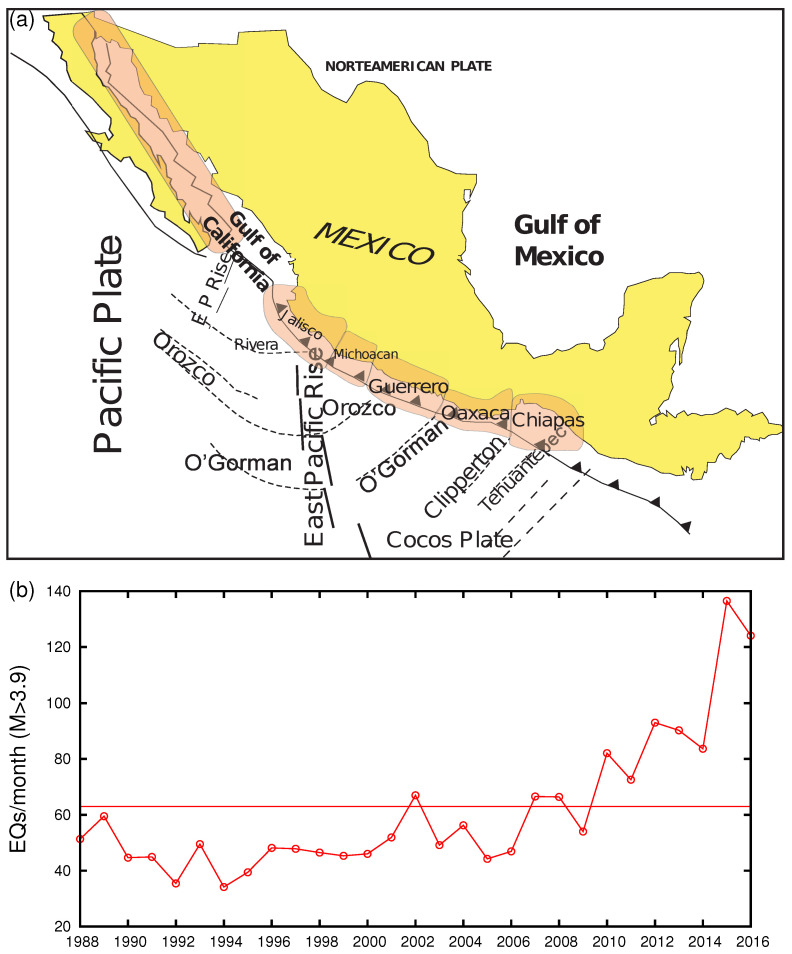
(color online) (**a**) Map of Mexico showing the six tectonic regions studied in natural time by Ramírez-Rojas and Flores-Márquez [39]. (**b**) The average monthly rate of M ≥4.0 EQs occurring allover Mexico, thus including all six regions shown in (**a**) from 1988 to 2016.

It is the scope of this paper to suggest a procedure to identify the occurrence time of the M8.2 EQ on 7 September 2017, which was Mexico’s largest earthquake in more than a century. This procedure is somewhat different from the one mentioned above applied in Reference [25] to the Tohoku EQ occurrence in 2011 in Japan. This is so because the latter procedure cannot be applied to the case of the M8.2 EQ in Mexico in 2017 since in this case neither the date of $\beta_{min}$ has been determined nor has the spatiotemporal study of $\beta_{min}$ been carried out to unveil an estimate of the epicentral area. To achieve our goal here, we used our results obtained in a previous paper [38] upon analyzing the seismicity in natural time, which showed that the occurrence of this M8.2 EQ in Mexico should not be considered unexpected. In particular, this earthquake occurred in the Chiapas region (CH) (see Figure 1a), where the probability for the occurrence of an extreme event was found by natural time analysis to be the highest compared to five other tectonic regions, i.e., Baja California (BC), Jalisco-Colima (J), Michoacán(M), Guerrero (G) and Oaxaca (O) of the Mexican Pacific Coast (Figure 1a), where the seismicity was studied in natural time by Ramírez-Rojas and Flores-Márquez [39] (see also Section 5). In other words, the candidate area of the impending mainshock has been estimated as being the Chiapas region. The average monthly rate for events M ≥4.0 occurring allover Mexico, thus including all six regions shown in Figure 1a, during the period from 1988 to 2016 is plotted in Figure 1b. This paper is structured as follows: In the next section, we explain in short the background of the general procedure followed here to determine the occurrence time of the impending mainschock, while in Section 3 we present the data analyzed. In Section 4, since SES data and/or Earth’s magnetic field anomalous variations have not yet been reported for this M8.2 EQ, and in order to identify at which date we should start the analysis of seismicity in natural time, we determine the date of the minimum $\beta_{min}$ of seismicity allover Mexico, which is expected as mentioned to be simultaneous with the initiation of the SES activity [30]. We then proceed in Section 5 to the determination of the occurrence time of the M8.2 Chiapas EQ following the procedure described in Section 2 making use of the condition $\kappa_{1}$ = 0.070. A short discussion follows in Section 6 and our conclusions are summarized in Section 7.

## 2. The Procedure to Determine the Occurrence Time of an Impending Mainshock. Background

Here, we follow the review in Reference [25]. In the time-series analysis using natural time, the behavior of the normalized power spectrum

(5) $$\Pi \left(\omega \right)\equiv {\left|\Phi \left(\omega \right)\right|}^{2}$$

defined by

(6) $$\Phi \left(\omega \right)=\sum_{k=1}^{N}p_{k}\exp\left(i\omega \chi_{k}\right)$$

where ω stands for the angular frequency at ω close to zero, is studied for capturing the dynamic evolution, because all the moments of the distribution of the $p_{k}$ can be estimated from Φ(ω) at ω→0 (see p. 499 of Reference [40]). For this purpose, a quantity $\kappa_{1}$ is defined from the Taylor expansion $\Pi \left(\omega \right)=1-\kappa_{1}\omega^{2}+\kappa_{2}\omega^{4}+\ldots$. The relation for the critical state that has been shown for SES activities [1,5]:

(7) $$\Pi \left(\omega \right)=\frac{18}{5\omega^{2}}-\frac{6\cos\omega }{5\omega^{2}}-\frac{12\sin\omega }{5\omega^{3}}.$$

for ω→0, simplifies to

(8) $$\Pi \left(\omega \right)\approx 1-0.07\omega^{2}$$

which shows that the second order Taylor expansion coefficient of Π(ω), i.e., $\kappa_{1}$, is equal to 0.070. This is also shown to be valid for earthquake models, e.g., for the time series of avalanches in the “train” Burridge–Knopoff earthquake model as well as in the Olami–Feder–Christensen earthquake model [5] when the system approaches the critical point.

Two procedures have been suggested in order to determine the occurrence time of the impending mainshock: A procedure, which for the sake of convenience has been called preliminary procedure, was initially followed (e.g., see References [1,22,41,42,43,44]) by starting the natural time analysis of seismicity in the candidate area *A* immediately after the SES initiation because the latter signals that the system enters the critical stage. Since the time variation of parameters were traced only on the single area *A*, a more objective procedure, called “updated” procedure, has been later developed [45] that considers the natural time analysis of the seismicity in *all* the possible subareas of *A*, instead of a single area. Here, we restricted ourselves, for the sake of simplicity, to the preliminary procedure. Beyond its simplicity, this procedure was applied here because its has already been shown [38,39] that the candidate epicentral area was the Chiapas region where the probability for the occurrence of an extreme event was found by natural time analysis to be the highest compared to five other tectonic regions in Mexico, as mentioned in the Introduction. The preliminary procedure could be summarized as follows [25]: We set the natural time zero at the initiation time of the SES activity, as mentioned, and then formed time series of seismic events in natural time for the area *A*, each time when a small EQ above a magnitude threshold $M\geq M_{thres}$ occured; in other words, when the number of the events increased by one. The normalized power spectrum in natural time Π(ω) for ω→0 (or the variance $\kappa_{1}$) for each of the time series was computed for the pair $\left(\chi_{k},p_{k}\right)$ and compared with that of Equation (7) for ω∈[0,π]. We also computed the two quantities *S* and $S_{-}$ defined in natural time as follows: The entropy *S* is defined as

(9) S≡〈χlnχ〉-〈χ〉ln〈χ〉

where the brackets $\langle \ldots \rangle \equiv \sum \left(\ldots \right)p_{k}$ denote averages with respect to the distribution $p_{k}$, i.e., $\langle f\left(\chi \right)\rangle \equiv \sum f\left(\chi_{k}\right)p_{k}$. Upon considering time reversal $\hat{T}$, i.e., $\hat{T}p_{k}=p_{N-k+1}$, the value *S* changes to a value $S_{-}$:

(10) $$S_{-}=\sum_{k=1}^{N}p_{N-k+1}\frac{k}{N}\ln\left(\frac{k}{N}\right)-\left(\sum_{l=1}^{N}\frac{l}{N}p_{N-l+1}\right)\ln\left[\sum_{k=1}^{N}\frac{k}{N}p_{N-k+1}\right]$$

In general, the value of $S_{-}$ is different from *S* and hence there exists a change $\Delta S\equiv S-S_{-}$ under time reversal. Thus, *S* does satisfy the condition to be “causal” [5,46]. The entropy *S* is a dynamic entropy that exhibits [47] concavity, positivity and Lesche stability [48,49]. The entropy $S_{u}$ of a uniform (*u*) distribution [5] is $S_{u}=0.096$.

The actual criteria for recognizing a *true* coincidence of the observed time series with that of critical state are as follows [1,5,34,41,42,43,44,50]:

First, the “average” distance 〈D〉 between the curves of Π(ω) of the evolving seismicity and Equation (7) should be smaller than 10${}^{-2}$.

Second, the final approach of the evolving Π(ω) to that of Equation (7) must be by descending from below, which alternatively means that before major EQs, the $\kappa_{1}$ value gradually changes with time and finally approaches from above that of the critical state, i.e., $\kappa_{1}=0.070$.

Third, both values *S* and $S_{-}$ should be smaller than $S_{u}$ at the coincidence.

Fourth, since the process concerned is supposed to be self-similar (critical dynamics), the time of the occurrence of the true coincidence should not vary, in principle, upon changing (within reasonable limits) the magnitude threshold $M_{thres}$ (and the size of area *A*).

It has been observed [1,41,42,43,44,51,52] that the aforementioned *true* coincidence appears usually a few days (up to around one week) before the occurrence of the mainshock.

## 3. Data and Analysis

**Figure 2:**
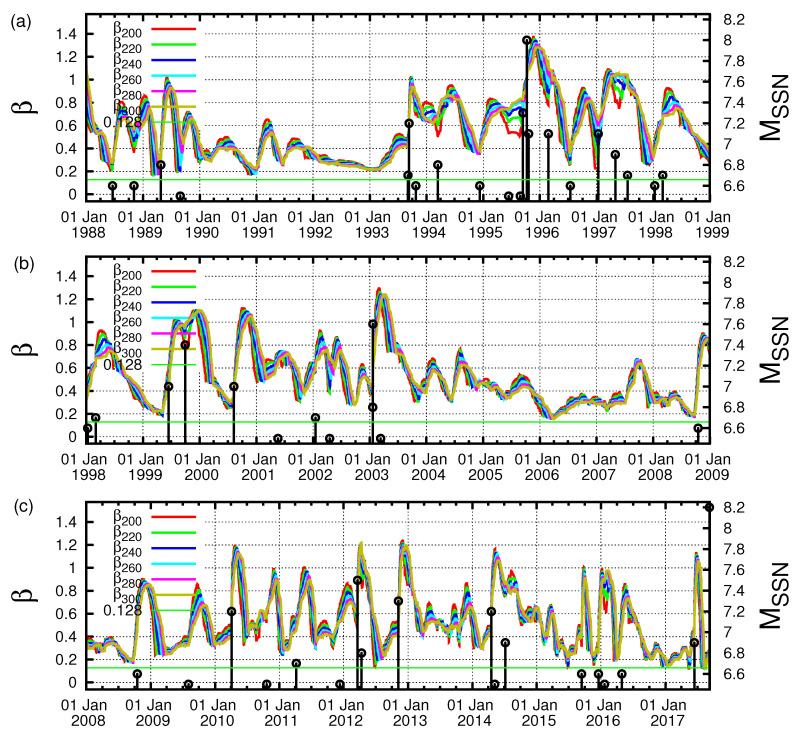
(color online) Plot of β values versus the conventional time at various scales *i* (number of events) for all M≥4.0 EQs in the entire Mexico region from 1 January 1988 until the M8.2 EQ occurrence: (**a**) from 1 January 1988 to 1 January 1999; (**b**) from 1 January 1999 to 1 January 2009; and (**c**) from 1 January 2009 to the M8.2 EQ occurrence on 7 September 2017. The black vertical lines ending at circles depict the EQ magnitudes that are read in the right hand scale.

**Figure 3:**
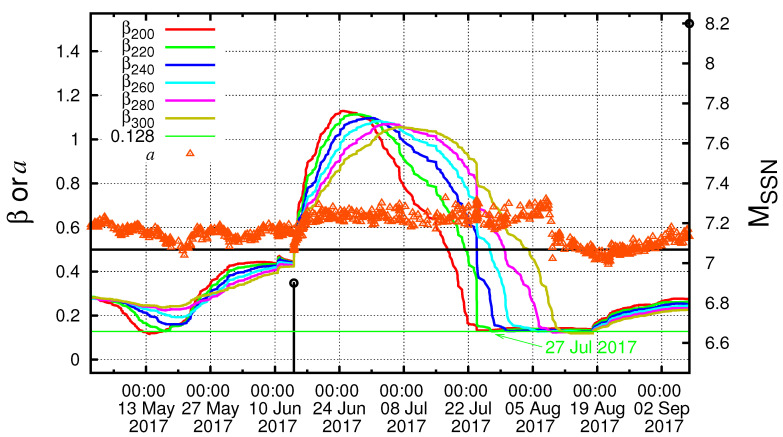
(color online)Plot of β values versus the conventional time at various scales *i* (number of events) for all M≥4.0 EQs in the entire Mexico region from 1 May 2017 until the M8.2 EQ occurrence. The $\beta_{min}$ shown with arrow was observed on 27 July during a period in which the DFA exponent *a* (orange triangles) was larger than 0.5. The black vertical lines ending at circles depict the EQ magnitudes which are read in the right hand scale.

The seismic data analyzed occurred allover Mexico, thus including all six regions depicted in Figure 1a covering an almost 30-year period, i.e., from 1 January 1988 until the M8.2 EQ occurrence on 7 September 2017. They came from the seismic catalog of the National Seismic Service (SSN) of the Universidad Nacional Autónoma de México (www.ssn.unam.mx). To assure catalog completeness, a magnitude threshold M≥4.0 was imposed. The average monthly rate during the period 1988–2016 is plotted as mentioned in Figure 1b. An inspection of this figure reveals that almost two years before the M8.2 EQ occurrence, the monthly rate was of the order of $10^{2}$ EQs/month, which is comparable to that in Japan. Thus, we studied the variability β for the same scales, i.e., from i=200 to 300 events, in a similar fashion as followed in our previous investigation in Japan (see Figures 2 and 3 of Reference [28]). These scales stand for the average number of M≥4.0 EQs occurring during the period of a few months, which corresponds to the average lead time of SES activities. This is not unreasonable if we recall that, according to the SES generation model (termed “pressure stimulated currents model” [5,53], see also References [32,54]), the SES activities are emitted when the EQ preparation area enters the critical stage.

Concerning the analysis, we applied the same procedure as in Reference [28], which could be summarized as follows: First, take an excerpt comprising *i* (≥100) successive EQs from the seismic catalogue. Then, form its sub-excerpts consisting of the *n*th to (*n* + 5)th EQs, (n=1,2,⋯,i-5) and compute $\kappa_{1}$ for each of them. In so doing, assign $\chi_{k}=k/6$ and the normalized energy $p_{k}=Q_{k}/\sum_{n=1}^{6}Q_{n}$, k=1,2,…,6 to the *k*th member of the sub-excerpt. Note that at least six EQs are needed for obtaining reliable $\kappa_{1}$. Iterate the same process for new sub-excerpts consisting of 7 members, 8 members, ⋯, and finally *i* members. Then, compute the average $\mu (\kappa_{1})$ and the standard deviation $\sigma (\kappa_{1})$ of thus obtained ensemble of (i-5)+(i-6)+…+1=(i-4)(i-5)/2 $\kappa_{1}$ values. The variability of $\kappa_{1}$ for this excerpt *i* (≥100) is defined to be $\beta \equiv \sigma \left(\kappa_{1}\right)/\mu \left(\kappa_{1}\right)$ (see Equation (4)) and is assigned to the (*i* + 1)th EQ, the target EQ. The time evolution of the β value can be pursued by sliding the excerpt through the EQ catalogue. Through the same process explained above, the values to be assigned to the (*i* + 2)th and (*i* + 3)th EQs in the catalogue are determined.

## 4. Determination of the Minimum of the Order Parameter Fluctuations of Seismicity

**Figure 4:**
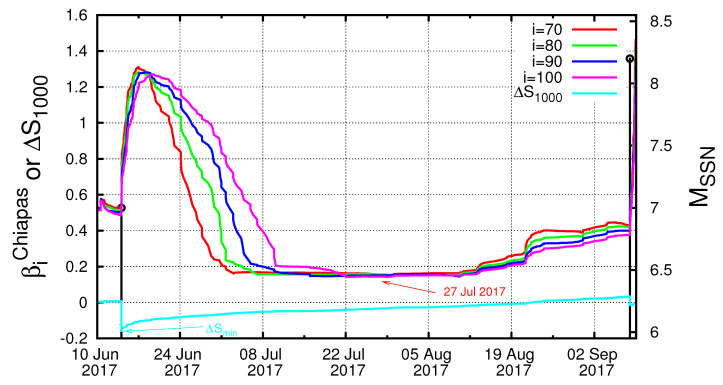
(color online) Plot of $\beta_{i}^{Chiapas}$ values versus the conventional time at various scales *i* (number of events) for all M≥4.0 EQs in the Chiapas region from 10 June 2017 until the M8.2 EQ occurrence. The minimum of $\beta_{i}^{Chiapas}$ for i=70 is shown with the red arrow and observed around 27 July. The cyan curve corresponds to $\Delta S_{i}$ for i=1000 as found in Reference [38] while the cyan arrow indicates the date of its minimum $\Delta S_{min}$ on 14 June 2017. The black vertical lines ending at circles depict the EQ magnitudes which are read in the right hand scale.

In Figure 2, we plot the β values computed in the entire region of Mexico for all M ≥4.0 EQs for various scales *i* (number of events) between i=200 and i=300 events versus the conventional time by starting the calculation on 1 January 1988, almost 30 years before the M8.2 Chiapas EQ on 7 September 2017. Earthquakes with M ≥6.5 (in the right hand scale) are shown by vertical bars ending at open circles. One can see in Figure 2 that β values fluctuate up and down so violently that it is hard to identify their correlations with EQs. However, one can notice that β shows a deep minimum value at scales lying between *i* = 200 and *i* = 300 events, i.e., for the scales i=220,240,260, and 280 events, just before the Chiapas EQ (see the rightmost side of the figure). This can be seen more clearly in Figure 3, which is an expanded version, in the conventional time, of the concerned part of Figure 2c, i.e., the almost four-month period from 1 May 2017 until the M8.2 EQ occurrence on 7 September 2017. An inspection of this figure reveals that the fluctuations β of the order parameter of seismicity was minimized on 27 July 2017, as marked with an arrow. This reflects that an SES activity initiated on 27 July 2017 when considering the findings in Reference [30] mentioned in the Introduction. Note that the date of $\beta_{min}$ computed in the entire Mexico region coincides with that resulting from the computation in Chiapas region shown in Figure 4 (such behavior is similar to the one observed in Japan [29]). The computed β values in Figure 4, which are labeled $\beta_{i}^{Chiapas}$ and are minimized around 27 July 2017 as marked with the red arrow, were calculated for the scales i=70,80,90, and 100 events, since the average number of EQs/month in the Chiapas region is almost three times smaller than that in the entire Mexico region.

## 5. Determination of the Occurrence Time of the M8.2 Earthquake

Concerning the starting time of the natural time analysis of seismicity, we chose the aforementioned date of 27 July 2017, which is the date of the appearance of $\beta_{min}$ of seismicity. As for the candidate area for the impending mainshock, although SES data have not yet been reported, we considered the Chiapas region as mentioned in the Introduction for the reasons explained in our previous paper [38], where we also used the results of the analysis of seismicity in the six areas of Mexico depicted in Figure 1a in natural time by Ramírez-Rojas and Flores-Márquez [39], which led to the identification of the properties (i.e., bimodal feature, non-Gaussian with a left exponential tail) of their probability density functions of the order parameter of seismicity pointing to an impending major EQ occurrence.

**Figure 5:**
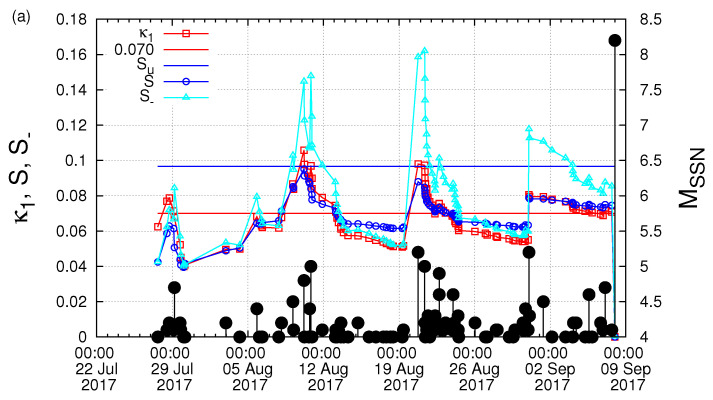

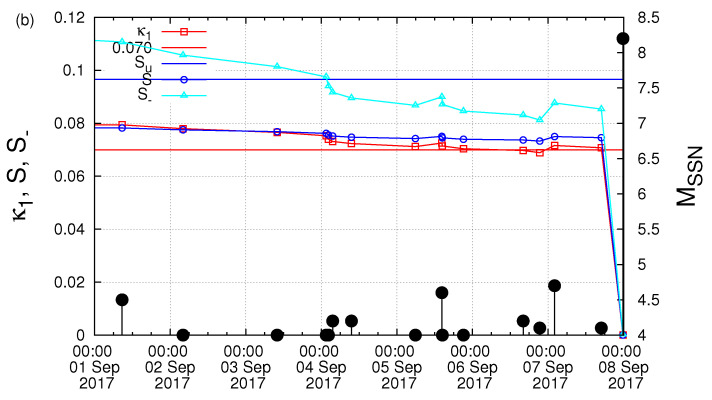
(color online) The evolution of the quantities $\kappa_{1}$, *S* and $S_{-}$ versus the conventional time by considering only the M ≥4.0 EQs that occurred inside the candidate epicentral area, i.e., the Chiapas region shown in Figure 1a: (**b**) an excerpt of (**a**) during the last week before the M8.2 EQ occurrence. The black vertical lines ending at circles depict the EQ magnitudes which are read in the right hand scale.

**Figure 6:**
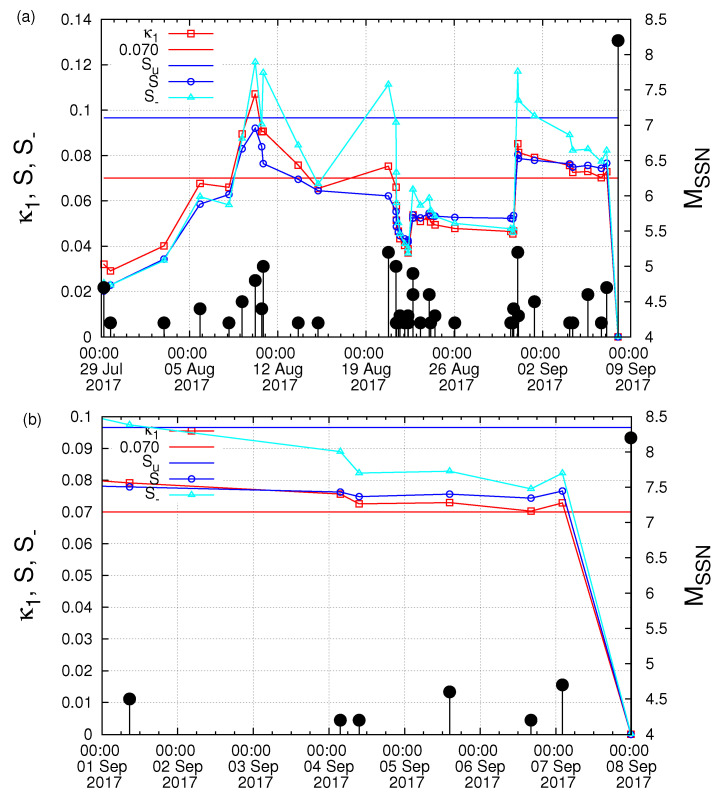
(color online) The evolution of the quantities $\kappa_{1}$, *S* and $S_{-}$ versus the conventional time by considering only the M ≥4.2 EQs that occurred inside the candidate epicentral area, i.e., the Chiapas region shown in Figure 1a: (**b**) an excerpt of (**a**) during the last week before the M8.2 EQ occurrence. The black vertical lines ending at circles depict the EQ magnitudes which are read in the right hand scale.

**Figure 7:**
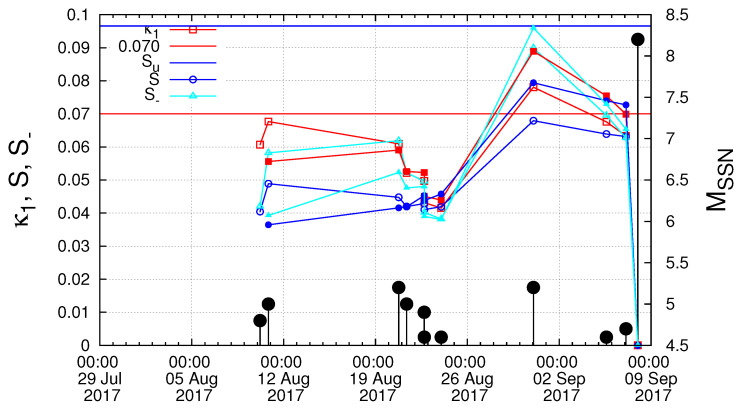
(color online) The evolution of the quantities $\kappa_{1}$ (red squares), *S* (blue circles) and $S_{-}$ (cyan triangles) versus the conventional time by considering only the M ≥4.6 EQs that occurred inside the candidate epicentral area, i.e., the Chiapas region shown in Figure 1a. The solid colored symbols correspond to the results obtained when the analysis in natural time starts on 10 August 2017, while the open ones when starting on 27 July 2017. The black vertical lines ending at circles depict the EQ magnitudes which are read in the right hand scale.

**Figure 8:**
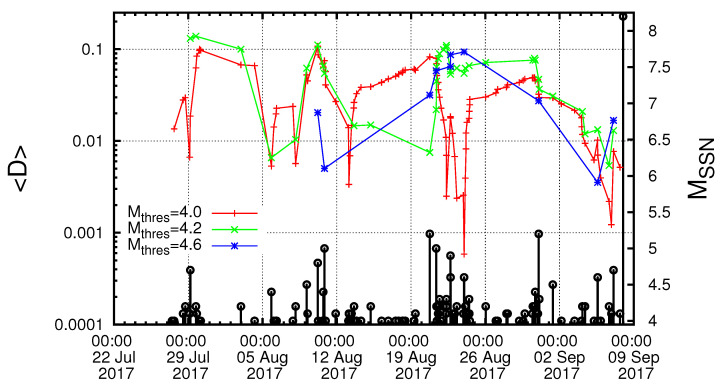
(color online) The evolution of the quantity “average” distance 〈D〉 between the curves of Π(ω) of the evolving seismicity and Equation (7) for various magnitude thresholds $M_{thres}=4.0$, 4.2 and 4.6. The black vertical lines ending at circles depict the EQ magnitudes, which are read in the right hand scale.

Starting from 27 July 2017, we next computed the $\kappa_{1}$ values of seismicity in the Chiapas region along with the corresponding values of *S* and $S_{-}$ and investigated the fulfilment of the four criteria that should be obeyed to characterize a true coincidence. The results are depicted in Figure 5 for $M_{thres}$= 4.0. A careful inspection of this figure reveals that the second and the third criteria explained in Section 2 to characterize as true the coincidence upon the occurrence of a M4.2 EQ at 16:06:25 UTC on 6 September 2017 were obeyed. The same behavior also held when repeating this calculation with other magnitude thresholds (as demanded by the fourth criterion concerning the magnitude threshold invariance explained in Section 2). For example, Figure 6, for $M_{thres}$ = 4.2, reveals that this behavior held upon the occurrence of the M4.2 EQ at 16:06:25 UTC on 6 September 2017. Another example is shown in Figure 7 for $M_{thres}$ = 4.6, in which we observed that the same behavior was obeyed upon the occurrence the M4.6 EQ at 14:17:38 UTC on 5 September 2017. Finally, we t checked the first criterion to be obeyed for a true coincidence explained in Section 2. In particular, as shown in Figure 8, we plotted the evolution of the values of the “average” distance 〈D〉 between the curves of Π(ω) of the evolving seismicity and Equation (7) for ω∈[0,π] and found that 〈D〉 was smaller than $10^{-2}$ (in all three magnitude thresholds mentioned above) a few days before the occurrence date of the M8.2 EQ on 7 September 2017. All previous occurrences of coincidences in Figure 8 were not valid for all magnitude thresholds.

Thus, in short, natural time analysis of seismicity in Chiapas region leads to the conclusion that the $\kappa_{1}$ values converged to 0.070 almost one day before the Chiapas earthquake occurrence, which pointed to the approach of the system to the critical point (mainshock) almost one day before the mainshock occurrence.

## 6. Discussion

In a previous paper [38], upon analyzing the seismicity of Chiapas region in natural time during the six-year period 2012–2017, we found that the entropy change ΔS under time reversal exhibited a pronounced minimum on 14 June 2017, i.e., almost three months before the occurrence of the M8.2 earthquake (see the cyan curve in Figure 4). Subsequently, we showed [55] that this ΔS minimum on 14 June 2017 was accompanied by precursory increase of the complexity measure Λ, defined [5,56] in natural time, which was associated with the fluctuations of the entropy change under time reversal (see also Reference [57]). Such a behavior of this complexity measure has been found to appear when a complex system approaches a dynamic phase transition as, for example, is the case of an impending sudden cardiac death risk ([58,59], see also subsection 9.4.1 of Reference [5]). Here, we showed that the minimum $\beta_{min}$ of the order parameter fluctuations happened later, i.e., on 27 July 2017. Such a sequence of dates is consistent with the one observed before the $M_{W}$9 Tohoku EQ that occurred in Japan on 11 March 2011. In particular, before the latter EQ, the entropy change under time reversal of the seismicity exhibited a minimum [60] on 22 December 2010, accompanied by a precursory increase of the Tsallis entropic index *q* [61,62,63] and almost two weeks later the $\beta_{min}$ of seismicity appeared on 5 January 2011 [28].

Furthermore, it is worth noting that, by applying Detrended Fluctuation Analysis (DFA) [64,65,66,67,68,69,70,71,72], which is a method that has been developed to address the problem of accurately quantifying long range correlations in non-stationary fluctuating signals to the earthquake magnitude time series, we found that the minimum $\beta_{min}$ of the fluctuations of the order parameter of seismicity was observed during a period in which long range correlations prevailed since the corresponding DFA exponent *a* was larger than 0.5 (see Figure 3), in a similar fashion with what happened before the aforementioned Tohoku EQ in Japan [73].

Our results presented in Section 5 were obtained upon considering that the date of $\beta_{min}$ is 27 July 2017. However, the analysis for various scales between i=200 and i=300 events showed a broad range of possible dates from 22 July to around 10 August 2017 (see Figure 3 and Figure 4). Hereafter, we discuss the influence of this uncertainty on the identification of the occurrence date of the impending major EQ. First, we started the computation of $\kappa_{1}$, *S* and $S_{-}$ values on 22 July 2017. For $M_{thres}=4.0$, we observed a coincidence upon the occurrence of the M4.0 EQ at 09:57:59 UTC on 3 September 2017. For $M_{thres}=4.2$, the results show two coincidences upon the occurrences of the M4.2 events at 09:34:10 UTC on 4 September 2017 and at 16:06:25 UTC on 6 September 2017. As for $M_{thres}=4.6$, we observed a coincidence upon the occurrence of the M4.6 EQ at 14:17:38 UTC on 5 September 2017. To summarize, if we started the computation on 22 July 2017, a coincidence may be considered as true if we assumed an uncertainty of around of a few days from 3 to 6 September 2017 for various magnitude thresholds. On the other hand, when we started the computation on 10 August 2017, we only observed (see the solid colored symbols in Figure 7) for $M_{thres}=4.6$ a coincidence upon the occurrence of the M4.7 EQ at 02:05:26 UTC on 7 September 2017, i.e., around 21 hours before the M8.2 EQ occurrence. Apparently, the uncertainty in starting date introduced some uncertainty in the exact moment/event at which the coincidence occurred, but the fact that we derived the coincidence remains.

## 7. Conclusions

Analyzing in natural time the seismicity of Mexico from 1 January 1988 until the occurrence of the M8.2 Chiapas EQ on 7 September 2017, and using sliding natural time window lengths comprising a number of events that would occur in a few months (which is comparable with the average value of the lead time of SES activities), the following results were obtained:

First, almost one and half months before the M8.2 Chiapas EQ, the minimum $\beta_{min}$ in the variability β of the order parameter of seismicity $\kappa_{1}$ was observed around 27 July 2017, which was the deepest during the almost 30 year period studied.

Second, starting from the above date of $\beta_{min}$ on 27 July 2017, which indicated that an SES activity initiated on this date, and computing the seismicity in the candidate area, i.e., in Chiapas area, we found that the condition $\kappa_{1}=0.070$ had been fulfilled (along with the fact that the criteria for recognizing a true coincidence were obeyed) on 6 September 2017.

In short, the procedure suggested here identified that the approach of the critical point (mainshock) happened almost one day before the M8.2 Chiapas EQ occurrence. 

## References

[1] Varotsos P.A., Sarlis N.V., Skordas E.S. (2001). Spatio-Temporal complexity aspects on the interrelation between Seismic Electric Signals and Seismicity. Pract. Athens Acad..

[2] Varotsos P.A., Sarlis N.V., Skordas E.S. (2002). Long-range correlations in the electric signals that precede rupture. Phys. Rev. E.

[3] Varotsos P.A., Sarlis N.V., Skordas E.S. (2003). Attempt to distinguish electric signals of a dichotomous nature. Phys. Rev. E.

[4] Varotsos P.A., Sarlis N.V., Skordas E.S. (2003). Long-range correlations in the electric signals the precede rupture: Further investigations. Phys. Rev. E.

[5] Varotsos P.A., Sarlis N.V., Skordas E.S. (2011). Natural Time Analysis: The New View of Time.

[6] Rundle J.B., Turcotte D.L., Donnellan A., Grant Ludwig L., Luginbuhl M., Gong G. (2016). Nowcasting earthquakes. Earth Space Sci..

[7] Rundle J.B., Luginbuhl M., Giguere A., Turcotte D.L. (2018). Natural Time, Nowcasting and the Physics of Earthquakes: Estimation of Seismic Risk to Global Megacities. Pure Appl. Geophys..

[8] Luginbuhl M., Rundle J.B., Hawkins A., Turcotte D.L. (2018). Nowcasting Earthquakes: A Comparison of Induced Earthquakes in Oklahoma and at the Geysers, California. Pure Appl. Geophys..

[9] Luginbuhl M., Rundle J.B., Turcotte D.L. (2018). Natural Time and Nowcasting Earthquakes: Are Large Global Earthquakes Temporally Clustered?. Pure Appl. Geophys..

[10] Rundle J.B., Giguere A., Turcotte D.L., Crutchfield J.P., Donnellan A. (2019). Global Seismic Nowcasting with Shannon Information Entropy. Earth Space Sci..

[11] Huang Q. (2008). Seismicity changes prior to the Ms8.0 Wenchuan earthquake in Sichuan, China. Geophys. Res. Lett..

[12] Huang Q. (2011). Retrospective investigation of geophysical data possibly associated with the Ms8.0 Wenchuan earthquake in Sichuan, China. J. Asian Earth Sci..

[13] Telesca L., Lovallo M. (2009). Non-uniform scaling features in central Italy seismicity: A non-linear approach in investigating seismic patterns and detection of possible earthquake precursors. Geophys. Res. Lett..

[14] Lennartz S., Livina V.N., Bunde A., Havlin S. (2008). Long-term memory in earthquakes and the distribution of interoccurrence times. EPL Europhys. Lett..

[15] Lennartz S., Bunde A., Turcotte D.L. (2011). Modelling seismic catalogues by cascade models: Do we need long-term magnitude correlations?. Geophys. J. Int..

[16] Rundle J.B., Holliday J.R., Graves W.R., Turcotte D.L., Tiampo K.F., Klein W. (2012). Probabilities for large events in driven threshold systems. Phys. Rev. E.

[17] Turcotte D.L. (1997). Fractals and Chaos in Geology and Geophysics.

[18] Carlson J.M., Langer J.S., Shaw B.E. (1994). Dynamics of earthquake faults. Rev. Mod. Phys..

[19] Holliday J.R., Rundle J.B., Turcotte D.L., Klein W., Tiampo K.F., Donnellan A. (2006). Space-Time Clustering and Correlations of Major Earthquakes. Phys. Rev. Lett..

[20] Botet R. (2011). Order parameter fluctuations at a critical point an exact result about percolation. J. Phys. Conf. Ser..

[21] Carretero-Campos C., Bernaola-Galván P., Ivanov P.C., Carpena P. (2012). Phase transitions in the first-passage time of scale-invariant correlated processes. Phys. Rev. E.

[22] Varotsos P.A., Sarlis N.V., Tanaka H.K., Skordas E.S. (2005). Similarity of fluctuations in correlated systems: The case of seismicity. Phys. Rev. E.

[23] Varotsos P., Sarlis N., Skordas E. (2011). Scale-specific order parameter fluctuations of seismicity in natural time before mainshocks. EPL Europhys. Lett..

[24] Kanamori H. (1978). Quantification of Earthquakes. Nature.

[25] Varotsos P.A., Sarlis N.V., Skordas E.S. (2017). Identifying the occurrence time of an impending major earthquake: A review. Earthq. Sci..

[26] Varotsos P., Sarlis N.V., Skordas E.S., Uyeda S., Kamogawa M. (2011). Natural time analysis of critical phenomena. Proc. Natl. Acad. Sci. USA.

[27] Sarlis N.V., Skordas E.S., Varotsos P.A. (2010). Order parameter fluctuations of seismicity in natural time before and after mainshocks. EPL Europhys. Lett..

[28] Sarlis N.V., Skordas E.S., Varotsos P.A., Nagao T., Kamogawa M., Tanaka H., Uyeda S. (2013). Minimum of the order parameter fluctuations of seismicity before major earthquakes in Japan. Proc. Natl. Acad. Sci. USA.

[29] Sarlis N.V., Skordas E.S., Varotsos P.A., Nagao T., Kamogawa M., Uyeda S. (2015). Spatiotemporal variations of seismicity before major earthquakes in the Japanese area and their relation with the epicentral locations. Proc. Natl. Acad. Sci. USA.

[30] Varotsos P.A., Sarlis N.V., Skordas E.S., Lazaridou M.S. (2013). Seismic Electric Signals: An additional fact showing their physical interconnection with seismicity. Tectonophysics.

[31] Varotsos P., Lazaridou M. (1991). Latest aspects of earthquake prediction in Greece based on Seismic Electric Signals. Tectonophysics.

[32] Varotsos P., Alexopoulos K., Lazaridou M. (1993). Latest aspects of earthquake prediction in Greece based on Seismic Electric Signals, II. Tectonophysics.

[33] Uyeda S., Hayakawa M., Nagao T., Molchanov O., Hattori K., Orihara Y., Gotoh K., Akinaga Y., Tanaka H. (2002). Electric and magnetic phenomena observed before the volcano-seismic activity in 2000 in the Izu Island Region, Japan. Proc. Natl. Acad. Sci. USA.

[34] Uyeda S., Kamogawa M., Tanaka H. (2009). Analysis of electrical activity and seismicity in the natural time domain for the volcanic-seismic swarm activity in 2000 in the Izu Island region, Japan. J. Geophys. Res..

[35] Xu G., Han P., Huang Q., Hattori K., Febriani F., Yamaguchi H. (2013). Anomalous behaviors of geomagnetic diurnal variations prior to the 2011 off the Pacific coast of Tohoku earthquake (Mw9.0). J. Asian Earth Sci..

[36] Han P., Hattori K., Xu G., Ashida R., Chen C.H., Febriani F., Yamaguchi H. (2015). Further investigations of geomagnetic diurnal variations associated with the 2011 off the Pacific coast of Tohoku earthquake (Mw 9.0). J. Asian Earth Sci..

[37] Han P., Hattori K., Huang Q., Hirooka S., Yoshino C. (2016). Spatiotemporal characteristics of the geomagnetic diurnal variation anomalies prior to the 2011 Tohoku earthquake (Mw 9.0) and the possible coupling of multiple pre-earthquake phenomena. J. Asian Earth Sci..

[38] Sarlis N.V., Skordas E.S., Varotsos P.A., Ramírez-Rojas A., Flores-Márquez E.L. (2018). Natural time analysis: On the deadly Mexico M8.2 earthquake on 7 September 2017. Physica A.

[39] Ramírez-Rojas A., Flores-Márquez E.L. (2013). Order parameter analysis of seismicity of the Mexican Pacific coast. Physica A.

[40] Feller W. (1971). An Introduction to Probability Theory and Its Applications, Vol. II.

[41] Varotsos P.A., Sarlis N.V., Skordas E.S., Tanaka H.K., Lazaridou M.S. (2006). Entropy of seismic electric signals: Analysis in the natural time under time reversal. Phys. Rev. E.

[42] Varotsos P.A., Sarlis N.V., Skordas E.S., Tanaka H.K., Lazaridou M.S. (2006). Attempt to distinguish long-range temporal correlations from the statistics of the increments by natural time analysis. Phys. Rev. E.

[43] Varotsos P. (2005). The Physics of Seismic Electric Signals.

[44] Varotsos P.A., Sarlis N.V., Skordas E.S., Lazaridou M.S. (2008). Fluctuations, under time reversal, of the natural time and the entropy distinguish similar looking electric signals of different dynamics. J. Appl. Phys..

[45] Sarlis N.V., Skordas E.S., Lazaridou M.S., Varotsos P.A. (2008). Investigation of seismicity after the initiation of a Seismic Electric Signal activity until the main shock. Proc. Jpn. Acad. Ser. B Phys. Biol. Sci..

[46] Varotsos P.A., Sarlis N.V., Skordas E.S., Lazaridou M.S. (2007). Identifying sudden cardiac death risk and specifying its occurrence time by analyzing electrocardiograms in natural time. Appl. Phys. Lett..

[47] Varotsos P.A., Sarlis N.V., Tanaka H.K., Skordas E.S. (2005). Some properties of the entropy in the natural time. Phys. Rev. E.

[48] Lesche B. (1982). Instabilities of Rényi entropies. J. Stat. Phys..

[49] Lesche B. (2004). Rényi entropies and observables. Phys. Rev. E.

[50] Varotsos P.A., Sarlis N.V., Skordas E.S. (2002). Seismic Electric Signals and Seismicity: On a tentative interrelation between their spectral content. Acta Geophys. Pol..

[51] Potirakis S.M., Asano T., Hayakawa M. (2018). Criticality Analysis of the Lower Ionosphere Perturbations Prior to the 2016 Kumamoto (Japan) Earthquakes as Based on VLF Electromagnetic Wave Propagation Data Observed at Multiple Stations. Entropy.

[52] Potirakis S.M., Schekotov A., Asano T., Hayakawa M. (2018). Natural time analysis on the ultra-low frequency magnetic field variations prior to the 2016 Kumamoto (Japan) earthquakes. J. Asian Earth Sci..

[53] Varotsos P., Alexopoulos K. (1986). Thermodynamics of Point Defects and Their Relation with Bulk Properties.

[54] Varotsos P. (2008). Point defect parameters in *β*-PbF_2_ revisited. Solid State Ion..

[55] Ramírez-Rojas A., Flores-Márquez E.L., Sarlis N.V., Varotsos P.A. (2018). The Complexity Measures Associated with the Fluctuations of the Entropy in Natural Time before the Deadly Mexico M8.2 Earthquake on 7 September 2017. Entropy.

[56] Sarlis N.V., Christopoulos S.R.G., Bemplidaki M.M. (2015). Change Δ*S* of the entropy in natural time under time reversal: Complexity measures upon change of scale. EPL Europhys. Lett..

[57] Sarlis N.V., Skordas E.S., Varotsos P.A., Ramírez-Rojas A., Flores-Márquez E.L. (2019). Investigation of the temporal correlations between earthquake magnitudes before the Mexico M8.2 earthquake on 7 September 2017. Physica A.

[58] Varotsos P.A., Sarlis N.V., Skordas E.S., Lazaridou M.S. (2004). Entropy in Natural Time Domain. Phys. Rev. E.

[59] Varotsos P.A., Sarlis N.V., Skordas E.S., Lazaridou M.S. (2005). Natural entropy fluctuations discriminate similar-looking electric signals emitted from systems of different dynamics. Phys. Rev. E.

[60] Sarlis N.V., Skordas E.S., Varotsos P.A. (2018). A remarkable change of the entropy of seismicity in natural time under time reversal before the super-giant M9 Tohoku earthquake on 11 March 2011. EPL Europhys. Lett..

[61] Varotsos P.A., Sarlis N.V., Skordas E.S. (2018). Tsallis Entropy Index q and the Complexity Measure of Seismicity in Natural Time under Time Reversal before the M9 Tohoku Earthquake in 2011. Entropy.

[62] Tsallis C. (1988). Possible generalization of Boltzmann-Gibbs statistics. J. Stat. Phys..

[63] Tsallis C. (2009). Introduction to Nonextensive Statistical Mechanics.

[64] Peng C.K., Buldyrev S.V., Goldberger A.L., Havlin S., Simons M., Stanley H.E. (1993). Finite-size effects on long-range correlations: Implications for analyzing DNA sequences. Phys. Rev. E.

[65] Peng C.K., Buldyrev S.V., Havlin S., Simons M., Stanley H.E., Goldberger A.L. (1994). Mosaic organization of DNA nucleotides. Phys. Rev. E.

[66] Buldyrev S.V., Goldberger A.L., Havlin S., Mantegna R.N., Matsa M.E., Peng C.K., Simons M., Stanley H.E. (1995). Long-range correlation properties of coding and noncoding DNA sequences: GenBank analysis. Phys. Rev. E.

[67] Taqqu M.S., Teverovsky V., Willinger W. (1995). Estimators for long-range dependence: An empirical study. Fractals.

[68] Talkner P., Weber R.O. (2000). Power spectrum and detrended fluctuation analysis: Application to daily temperatures. Phys. Rev. E.

[69] Hu K., Ivanov P.C., Chen Z., Carpena P., Stanley H.E. (2001). Effect of trends on detrended fluctuation analysis. Phys. Rev. E.

[70] Chen Z., Ivanov P.C., Hu K., Stanley H.E. (2002). Effect of nonstationarities on detrended fluctuation analysis. Phys. Rev. E.

[71] Chen Z., Hu K., Carpena P., Bernaola-Galvan P., Stanley H.E., Ivanov P.C. (2005). Effect of nonlinear filters on detrended fluctuation analysis. Phys. Rev. E.

[72] Xu L., Ivanov P.C., Hu K., Chen Z., Carbone A., Stanley H.E. (2005). Quantifying signals with power-law correlations: A comparative study of detrended fluctuation analysis and detrended moving average techniques. Phys. Rev. E.

[73] Varotsos P.A., Sarlis N.V., Skordas E.S. (2014). Study of the temporal correlations in the magnitude time series before major earthquakes in Japan. J. Geophys. Res. Space Phys..

